# Ultrasound-Based Morphofunctional Assessment of the Transversus Abdominis and Urinary Incontinence in Female Rugby Players

**DOI:** 10.3390/jfmk11030284

**Published:** 2026-07-21

**Authors:** Sheila Fernández García, Sara Cerrolaza-Tudanca, Eva María Rodríguez-González, María Soledad Amor-Salamanca, Ivan Herrera-Peco, Manuel Rozalén-Bustín

**Affiliations:** 1 Facultad de Ciencias Biomédicas y de la Salud, Universidad Alfonso X el Sabio, Villanueva de la Cañada, 28692 Madrid, Spain; 2 Facultad HM de Ciencias de la Salud, Universidad Camilo José Cela, Villanueva de la Cañada, 28692 Madrid, Spain; 3 Instituto de Investigación Sanitaria HM Hospitales, 28015 Madrid, Spain; 4 Clínicas Dr. Rozalén, Calle O’Donnell 49, 28009 Madrid, Spain

**Keywords:** urinary incontinence, female athletes, functional morphology, pelvic floor, rugby, transversus abdominis, rehabilitative ultrasound imaging

## Abstract

**Background:** Female rugby players are exposed to repeated contact, impact, acceleration, deceleration, and trunk-stabilization demands that may challenge lumbopelvic control and intra-abdominal pressure regulation. Rehabilitative ultrasound imaging can characterize transversus abdominis (TrA) thickness changes during standardized tasks, although these measures should not be interpreted as direct indicators of neuromuscular activation or sport-specific trunk function. This study aimed to characterize ultrasound-derived TrA thickness and thickness change in nulliparous female rugby players and to explore whether these measures, immediate visual feedback, sport-related variables, and body mass index were associated with urinary leakage. **Methods:** A cross-sectional observational study was conducted with 34 nulliparous female rugby players recruited from two competitive rugby clubs. Urinary leakage was assessed using the validated Spanish version of the International Consultation on Incontinence Questionnaire-Short Form (ICIQ-UI SF), with current urinary leakage defined as leakage reported within the four-week recall period. TrA thickness was measured bilaterally using rehabilitative ultrasound imaging at rest, during the abdominal drawing-in maneuver (ADIM), and during ADIM with real-time ultrasound-based visual feedback. Exploratory analyses were conducted to examine associations between current urinary leakage and ultrasound-derived TrA measures, sport-related variables, and body mass index. **Results:** Overall, 41.2% of participants reported having experienced urinary leakage at some point, while 20.6% reported current urinary leakage. Among participants reporting leakage type, stress urinary incontinence was the most frequent pattern, and perceived impact on daily life was low. TrA thickness increased bilaterally during ADIM compared with rest. Immediate visual feedback did not produce a statistically significant additional increase in TrA thickness. No significant associations were observed between current urinary leakage and TrA thickness measures, side-to-side differences, weekly training hours, years of rugby practice, or body mass index. **Conclusions:** In this exploratory sample, ultrasound-derived TrA thickness measures during controlled ADIM tasks were not significantly associated with current urinary leakage. Future studies should include larger samples, direct pelvic floor muscle assessment, respiratory monitoring, body composition measures, and dynamic rugby-specific tasks.

## 1. Introduction

Rugby is a high-intensity contact sport that requires repeated acceleration, deceleration, tackling, sprinting, jumping, changes in direction, and sustained trunk stabilization. These sport-specific demands place considerable mechanical load on the lumbopelvic region, where the abdominal wall and pelvic floor muscles contribute to spinal stability, force transmission, postural control, and regulation of intra-abdominal pressure (IAP) [1]. As women’s participation in rugby continues to increase, there is growing interest in understanding how the morphological characteristics and ultrasound-derived behavior of the deep trunk musculature may relate to pelvic health, continence mechanisms, and sports rehabilitation needs.

Urinary incontinence (UI), defined as any involuntary loss of urine through the urethra, is a relevant but often underreported pelvic health concern among physically active women and female athletes [2,3,4]. In high-impact and contact sports, repetitive elevations in IAP may exceed the capacity of the pelvic floor muscles (PFM) to maintain urethral closure, thereby contributing to stress urinary incontinence (SUI) [5,6]. SUI is considered the most common form of UI among women participating in high-impact activities such as rugby, athletics, and artistic gymnastics [7,8]. Previous studies have reported relevant UI prevalence in female athletes, with higher rates in sports characterized by jumping, landing, rapid directional changes, and repeated loading of the pelvic floor [2,9,10,11,12]. These rates contrast with those observed in non-athletic women and highlight the need to consider UI as a sports medicine and rehabilitation issue rather than only as a urogynaecological condition [7]. Studying nulliparous athletes is particularly relevant because it reduces the potential confounding effect of pregnancy and childbirth, two well-established contributors to pelvic floor dysfunction, and allows sport-related pelvic floor loading to be examined in a more specific athletic context.

From a functional morphology perspective, continence during sport depends not only on the structural capacity of the PFM but also on the coordinated behavior of the abdominal wall, diaphragm, and pelvic floor during tasks that increase IAP. The transverse abdominis muscle (TrA), the deepest layer of the abdominal wall, plays an important role in lumbopelvic stabilization and anticipatory trunk control [13]. Its coordinated contraction with the PFM may contribute to pelvic stability and continence during high-load activities [14,15,16]. However, although rugby players may present well-developed trunk musculature due to the physical requirements of the sport, greater muscle thickness does not necessarily imply optimal neuromuscular timing, pressure management, or TrA–PFM synergy [17]. This distinction is particularly relevant in functional morphology and kinesiology, where muscle structure, activation capacity, and task-specific coordination should be interpreted together. Nevertheless, TrA–PFM coordination cannot be inferred from TrA thickness alone and requires direct or complementary assessment of pelvic floor muscle function. Rehabilitative ultrasound imaging has become a useful non-invasive tool for assessing abdominal muscle morphology and thickness changes during standardized contraction tasks in clinical and sports rehabilitation settings [18]. Compared with more invasive or costly imaging techniques, ultrasound allows real-time visualization of muscle thickness during standardized tasks such as the abdominal drawing-in maneuver (ADIM), which is commonly used to assess selective TrA thickening under controlled low-load conditions [8,19]. Previous studies have suggested that women with UI may show altered TrA thickness responses during ADIM compared with continent women, and that reduced TrA thickening may reflect impaired deep trunk muscle behavior [9,20,21]. However, most available evidence has focused on non-athletic women or athletes from sports such as gymnastics and athletics, while rugby players remain comparatively underexplored despite the sport’s substantial lumbopelvic and pelvic floor demands [22,23].

An important conceptual distinction should be made between ADIM and abdominal bracing. ADIM is a low-load motor task designed to preferentially recruit the deep abdominal wall, particularly the TrA, and is useful for standardized ultrasound assessment. In contrast, abdominal bracing involves a more global co-contraction strategy of the abdominal wall and is likely more representative of the trunk stiffness required during high-load rugby actions such as tackling, contact preparation, sprinting, and rapid changes in direction [24]. Therefore, ADIM-derived TrA thickness changes should not be interpreted as direct evidence of rugby-specific trunk stabilization.

The ADIM is also frequently incorporated into physiotherapy protocols aimed at improving deep abdominal muscle awareness and lumbopelvic control [25]. Real-time ultrasound feedback may enhance motor learning by helping athletes visualize muscle thickening during contraction attempts and improve task execution [6,10]. Although biofeedback has been used in pelvic floor rehabilitation and has been proposed as a strategy to improve muscle awareness and reduce UI symptoms, its immediate effect on TrA thickness change during ADIM in trained rugby players remains unclear [26]. Because athletes may already have high baseline neuromuscular control, the usefulness of visual feedback may depend on whether the assessment is performed in static, semi-static, or dynamic sport-specific conditions.

Several sport-related and individual factors may also influence UI risk in female athletes. Weekly training hours, accumulated years of sport participation, training intensity, and technical execution have been proposed as potential contributors to pelvic floor overload [15,25,27]. Body mass index (BMI) has also been associated with UI in the general population, although its interpretation in athletes is complex because higher BMI may reflect greater muscle mass rather than excess adiposity, particularly in rugby players [13,20,28,29]. Therefore, studies in female rugby players should consider both anthropometric and sport-specific variables while acknowledging that static measures may not fully capture functional continence mechanisms during high-impact tasks.

UI also has psychological, social, and participation-related implications for athletes. Feelings of embarrassment, stigma, lack of information, and normalization of symptoms may lead female athletes to underreport UI, modify training behavior, or avoid seeking professional support [21,30]. For this reason, pelvic health assessment in women’s rugby should be integrated into a broader sports rehabilitation and athlete-monitoring framework that includes education, functional screening, and individualized preventive strategies.

Despite increasing interest in UI among female athletes, limited research has specifically examined the relationship between TrA morphology, ultrasound-derived TrA thickness response, visual feedback, and continence status in rugby players. Addressing this gap may help clarify whether static and semi-static ultrasound measures of TrA thickness provide clinically useful information for pelvic health monitoring in this population, or whether future assessments should prioritize dynamic coordination between the TrA and PFM during sport-specific tasks. However, because the PFM function was not directly assessed in the present study, any reference to TrA–PFM coordination should be interpreted as a mechanistic framework rather than as a measured outcome.

Therefore, this study aimed to characterize ultrasound-derived TrA thickness and change in thickness using rehabilitative ultrasound imaging in nulliparous female rugby players. Specifically, TrA thickness was assessed bilaterally at rest, during the ADIM task, and during the ADIM task with real-time ultrasound-based visual feedback. A secondary aim was to describe the prevalence and characteristics of urinary leakage in this athletic population and to explore whether static and semi-static ultrasound-derived TrA measures, immediate visual-feedback-related changes, sport-related variables, and body mass index were associated with current or previous urinary leakage. This approach was intended to clarify whether ultrasound-derived TrA thickness provides relevant information for pelvic health assessment and athlete monitoring in women’s rugby, while acknowledging that ADIM does not reproduce the bracing strategy or dynamic loading conditions required during rugby-specific actions.

## 2. Materials and Methods

### 2.1. Study Design

This observational, descriptive, cross-sectional study was designed to examine ultrasound-derived transversus abdominis (TrA) thickness and thickness changes in nulliparous female rugby players using rehabilitative ultrasound imaging. The study focused on TrA thickness under three standardized assessment conditions: at rest, during the abdominal drawing-in maneuver (ADIM) task, and during ADIM performance with real-time ultrasound-based visual feedback.

The study was exploratory in nature. Static and semi-static ultrasound-derived TrA measures, side-to-side thickness differences, feedback-related thickness changes, sport-related variables, and body mass index were analyzed in relation to current and previous urinary leakage. The study followed the Strengthening the Reporting of Observational Studies in Epidemiology (STROBE) recommendations for cross-sectional studies.

### 2.2. Participants and Recruitment

A convenience sample of 34 nulliparous female rugby players was recruited from two competitive rugby clubs. Recruitment and data collection were conducted between May and June 2025.

Participants were eligible if they were female rugby players, nulliparous, aged 18 years or older, had practiced rugby regularly during the previous six months, and trained for at least two hours per week. The exclusion criteria were neuromuscular disorders, neurological disorders, connective tissue disorders, active rheumatological disease, systemic disease, previous abdominal hernia or abdominal surgery, orthopedic surgery involving the lumbar spine, pelvis, or lower limbs during the previous six months, and pregnancy.

Recruitment was based on player availability during the study period. No formal a priori sample size calculation was performed because this was an exploratory cross-sectional study conducted in a specific and difficult-to-access athletic population. The final sample size was determined by player availability during the recruitment period. The number of players initially approached and the reasons for non-participation, were not systematically recorded; therefore, a detailed participant flow diagram could not be reconstructed. All participants received information about the study objectives and procedures before data collection and provided written informed consent.

### 2.3. Sociodemographic, Clinical, and Sport-Related Variables

Sociodemographic, anthropometric, clinical, and sport-related data were collected using a structured questionnaire. The variables recorded included age, weight, height, body mass index, educational level, employment status, smoking status, constipation status, family history of urinary incontinence, reproductive system anomalies, history of gynecological surgery, urinary tract infection during the previous month, weekly training hours, and years of rugby practice.

Body mass index was calculated as weight in kilograms divided by height in meters squared. Training exposure was assessed using two variables: number of training hours per week and accumulated years of rugby practice.

Playing position, match exposure, number of weekly matches, tackle/contact exposure, strength training load, previous pelvic floor physiotherapy, menstrual status, hormonal contraceptive use, and previous sports participation were not systematically recorded. Therefore, these variables could not be included in the present analysis and were considered as relevant limitations for the interpretation of sport-specific pelvic floor loading.

### 2.4. Urinary Incontinence Assessment

Urinary leakage was assessed using the validated Spanish version of the International Consultation on Incontinence Questionnaire-Short Form (ICIQ-UI SF) [31]. The questionnaire includes items assessing leakage frequency, amount of leakage, perceived impact on daily life, and situations in which urinary leakage occurs. Participants were asked to report their urinary leakage experiences during the previous four weeks.

For the purposes of the present study, urinary leakage was described using two complementary variables: history of urinary leakage at any time and current urinary leakage. History of urinary leakage at any time was obtained from a complementary self-report item included in the study questionnaire. Current urinary leakage was defined as urinary leakage reported within the four-week recall period of the ICIQ-UI SF. Urinary incontinence subtype was classified according to the reported leakage situation as stress urinary incontinence, urgency urinary incontinence, or mixed urinary incontinence.

The perceived impact of urinary leakage on daily life was assessed using the quality-of-life impact item of the ICIQ-UI SF, scored from 0 to 10, with higher values indicating greater perceived impact.

### 2.5. Rehabilitative Ultrasound Imaging

TrA thickness was assessed using a CHISON SonoEye P2 portable ultrasound device (CHISON Medical Technologies Co., Ltd., Wuxi, Jiangsu, China) equipped with a 7.5 MHz linear probe. The ultrasound device was used in B-mode. The frequency was adjusted to 10 MHz for superficial muscle imaging, and the scanning depth was generally set at 5 cm. In participants with greater subcutaneous tissue thickness, the depth was increased to 6 cm to optimize visualization of the deep abdominal muscle interface.

TrA thickness was measured on the ultrasound image as the distance between the superficial and deep fascial borders of the TrA, using a line perpendicular to the muscle fascial planes at the selected measurement point. The measurement point was kept consistent across resting, ADIM, and visual-feedback conditions for each participant. Ultrasound-derived TrA thickness and thickness change were interpreted as structural responses during standardized tasks and not as direct measures of neuromuscular activation, force production, or muscle function.

All ultrasound assessments were performed by the same physiotherapist during the same data collection period. Measurements were obtained bilaterally, first on the right side and then on the left side. Three measurements were recorded for each condition, and the mean of the three measurements was used for statistical analysis.

### 2.6. Participant Positioning and Probe Placement

Participants were positioned in supine lying on an examination table, with approximately 45° of hip flexion and 90° of knee flexion. The soles of the feet remained in contact with the table, the lumbar spine was kept in contact with the surface, the pelvis was maintained in a neutral position, and the upper limbs were placed alongside the body.

The TrA was assessed on the anterolateral abdominal wall. The lower border of the rib cage and the anterior superior iliac crest were used as anatomical landmarks. The transducer was placed transversely midway between these two landmarks, following the anterior axillary line. The probe position was adjusted to clearly identify the abdominal wall layers, including the external oblique, internal oblique, and TrA. The same anatomical location and probe orientation were used for all measurements on both sides. Care was taken to avoid excessive probe pressure that could deform the abdominal wall and affect thickness measurements.

### 2.7. Ultrasound Assessment Conditions

TrA thickness was assessed under three standardized conditions.

First, resting TrA thickness was measured with the participant in the initial supine position. The ultrasound image was captured at the end of a relaxed expiration.

Second, TrA thickness was measured during the ADIM task. Participants were instructed to perform one normal breathing cycle followed by a forced expiration. After exhaling, they were asked to draw the lower abdomen inward and upward toward the spine, without pelvic movement or visible compensatory strategies. The contraction was maintained for approximately five seconds. The ultrasound image was captured at the point of greatest abdominal drawing-in during the end-expiratory/apnea phase.

Third, TrA thickness was measured during ADIM with real-time ultrasound-based visual feedback. In this condition, participants were allowed to observe the ultrasound image on the screen while identifying the TrA at rest and during the contraction attempt. They were then instructed to perform the ADIM while using the ultrasound image as visual feedback. Image capture was performed at the same phase of the maneuver as in the non-feedback ADIM condition. The ADIM was selected as a standardized low-load ultrasound assessment task and was not intended to reproduce abdominal bracing or high-load trunk stabilization during rugby-specific actions. Respiratory flow, breath-holding duration, and intra-abdominal pressure were not instrumentally monitored.

For each condition, TrA thickness was recorded separately on the right and left sides. Side-to-side differences were calculated to explore bilateral asymmetry. Rest-to-ADIM thickness changes were calculated to describe ultrasound-derived thickening during the task. The visual-feedback effect was explored by comparing TrA thickness during ADIM without visual feedback and ADIM with visual feedback.

### 2.8. Study Outcomes

The primary morphofunctional outcomes were right and left TrA thickness at rest, during ADIM, and during the ADIM task with real-time ultrasound-based visual feedback.

Derived ultrasound variables included side-to-side TrA thickness difference, rest-to-ADIM thickness change, and feedback-related thickness change. These variables were used to explore whether bilateral asymmetry, activation-related thickening, or immediate visual-feedback-related thickness changes were associated with current urinary leakage.

The primary clinical outcome was current urinary leakage, defined as urinary leakage reported within the four-week recall period of the ICIQ-UI SF. Secondary clinical outcomes included history of urinary leakage at any time, leakage frequency, leakage amount, urinary incontinence subtype, and perceived impact on daily life.

Sport-related outcomes included weekly training hours and years of rugby practice.

### 2.9. Ethical Considerations

The study was conducted in accordance with the ethical principles of the Declaration of Helsinki. The study protocol was approved by the Ethics Committee of Universidad Alfonso X el Sabio (2025_01/334, approved date: 27 January 2025). All participants received information about the study objectives, procedures, potential risks, and benefits, and all provided written informed consent before participation. Confidentiality and data protection were ensured in accordance with current personal data protection regulations.

### 2.10. Statistical Analysis

Data were analyzed using Stata 16.0 statistical software (StataCorp, College Station, TX, USA). Descriptive statistics were calculated for all study variables. Quantitative variables were summarized using means, standard deviations, minimum values, and maximum values. Categorical variables were summarized using absolute and relative frequencies. Percentages were calculated using the available denominator for each variable, and missing data were reported when present.

The distribution of quantitative variables was assessed using the Shapiro–Wilk test. Homogeneity of variance was examined using Levene’s test when appropriate. Comparisons of TrA thickness between the right and left sides and between assessment conditions were performed using paired *t*-tests or non-parametric alternatives according to data distribution.

Comparisons between participants with and without current urinary incontinence were conducted using Student’s *t*-test or the Mann–Whitney U test for quantitative variables and Pearson’s chi-square test or Fisher’s exact test for categorical variables, as appropriate.

Associations between current urinary leakage and ultrasound-derived, anthropometric, clinical, and sport-related variables were explored using bivariate analyses. These variables included TrA thickness measures, side-to-side thickness differences, feedback-related thickness changes, body mass index, weekly training hours, years of rugby practice, smoking status, constipation status, and relevant clinical history variables.

When logistic regression analyses were performed, odds ratios and 95% confidence intervals were reported. Odds ratios for TrA thickness variables were based on the original centimeter scale and were therefore interpreted cautiously, given the small range of observed values and the exploratory nature of the analyses.

Multivariable regression modeling was not conducted because of the exploratory nature of the study, the modest sample size, and the limited number of participants with current urinary leakage. Results were interpreted as exploratory cross-sectional associations and not as evidence of causal or predictive effects. All statistical tests were two-sided, and statistical significance was set at *p* < 0.05.

## 3. Results

### 3.1. Participant Characteristics

The study included 34 nulliparous female rugby players. Participants had a mean age of 26.3 ± 5.0 years, with an age range from 18.9 to 37.4 years. Regarding anthropometric characteristics, mean body weight was 68.0 ± 10.4 kg, mean height was 1.64 ± 0.05 m, and mean body mass index (BMI) was 25.2 ± 3.7 kg/m^2^. From a sport-related perspective, participants trained an average of 6.8 ± 3.2 h per week and had 7.4 ± 5.2 years of accumulated rugby experience (Table 1).

Most participants had university-level education (79.4%). Regarding clinical and lifestyle-related variables, 79.4% were non-smokers, 14.7% reported constipation, and 11.8% reported occasional constipation. A family history of urinary incontinence was reported by 17.7% of participants, while 32.4% were unsure. Reproductive system anomalies were reported by 5.9% of participants, previous gynecological surgery by 11.8%, and urinary tract infection in the previous month by 2.9% (Table 2).

### 3.2. Urinary Leakage Characteristics

Overall, 14 participants (41.2%) reported having experienced urinary leakage at some point, while 7 participants (20.6%) reported current urinary leakage at the time of the study.

Among participants with current urinary leakage, 6 reported leakage 1–3 times per week and 1 reported leakage once per day. Regarding leakage amount, 6 participants reported a very small amount of urine loss, and 1 reported a moderate amount.

Among participants who reported the type of urinary leakage, stress urinary incontinence was the most frequent pattern, reported by 9 participants (69.2%), followed by mixed urinary incontinence in 3 participants (23.1%) and urgency urinary incontinence in 1 participant (7.7%). The perceived impact of urinary leakage on daily life was low; 27 participants rated the impact as 1 out of 10, 5 rated it as 2, 1 rated it as 3, and 1 rated it as 4 (Table 3).

### 3.3. Morphofunctional Ultrasound Assessment of Transversus Abdominis Thickness

TrA thickness was assessed bilaterally under three standardized conditions: at rest, during voluntary activation using the abdominal drawing-in maneuver (ADIM), and during ADIM with real-time ultrasound-based visual feedback.

At rest, TrA thickness was slightly greater on the left side than on the right side. Mean resting thickness was 0.42 ± 0.08 cm on the right side and 0.46 ± 0.10 cm on the left side. During ADIM without visual feedback, TrA thickness increased on both sides, with mean values of 0.84 ± 0.19 cm on the right side and 0.80 ± 0.19 cm on the left side. During ADIM with visual feedback, TrA thickness was 0.86 ± 0.19 cm on the right side and 0.85 ± 0.21 cm on the left side.

The pattern of thickness change indicated an ADIM-related increase in TrA thickness from rest on both sides. Mean right-left differences were small across conditions: −0.03 ± 0.07 cm at rest, 0.04 ± 0.18 cm during ADIM without feedback, and 0.01 ± 0.16 cm during ADIM with visual feedback. The resting right-left difference was statistically significant, with greater resting thickness on the left side, whereas right-left differences during ADIM without feedback and ADIM with visual feedback were not statistically significant (Table 4).

### 3.4. ADIM-Related Thickness Changes and Immediate Visual Feedback Effect

The increase in TrA thickness from rest to activation was observed bilaterally. On the right side, mean TrA thickness increased from 0.42 ± 0.08 cm at rest to 0.84 ± 0.19 cm during ADIM and 0.86 ± 0.19 cm during ADIM with visual feedback. On the left side, mean TrA thickness increased from 0.46 ± 0.10 cm at rest to 0.80 ± 0.19 cm during ADIM and 0.85 ± 0.21 cm during ADIM with visual feedback.

When ADIM without visual feedback was compared with ADIM with visual feedback, no statistically significant differences were observed. The mean difference between ADIM with and without feedback was 0.018 cm on the right side (95% CI: −0.059 to 0.096; *p* = 0.639) and 0.041 cm on the left side (95% CI: −0.042 to 0.124; *p* = 0.327). Therefore, although TrA thickness increased during the ADIM task, immediate ultrasound-based visual feedback did not produce a statistically significant additional increase in TrA thickness (Table 5).

### 3.5. Association Between Ultrasound-Derived TrA Measures and Urinary Leakage

Exploratory analyses were conducted to examine whether ultrasound-derived TrA measures were associated with current urinary leakage. No statistically significant association was observed between right-left TrA thickness difference and current urinary leakage (OR = 0.28; 95% CI: 0.01–9.55; *p* = 0.481).

Similarly, current urinary leakage was not significantly associated with right TrA thickness (OR = 0.58; 95% CI: 0.09–3.88; *p* = 0.574) or left TrA thickness (OR = 0.84; 95% CI: 0.12–6.07; *p* = 0.862). These findings suggest that, in this exploratory sample, static and semi-static ultrasound-derived TrA thickness measures were not significantly associated with current urinary leakage.

Exploratory analyses using history of urinary leakage as the outcome showed a statistically significant association with right-left TrA thickness difference (OR = 27.59; 95% CI: 1.43–530.80; *p* = 0.028). However, this finding should be interpreted cautiously because of the small sample size and the very wide confidence interval. Left TrA thickness showed a near-threshold association with history of urinary leakage (OR = 0.19; 95% CI: 0.03–1.06; *p* = 0.058), whereas right TrA thickness was not significantly associated with this outcome (OR = 0.65; 95% CI: 0.14–3.06; *p* = 0.587) (Table 6).

### 3.6. Association Between Sport-Related Variables, BMI, and Urinary Leakage

Weekly training hours and years of rugby practice were not significantly associated with current urinary leakage. Specifically, no significant association was observed between current urinary leakage and weekly training hours (OR = 0.89; 95% CI: 0.64–1.23; *p* = 0.482) or accumulated years of rugby practice (OR = 0.96; 95% CI: 0.80–1.15; *p* = 0.651). BMI was also not significantly associated with current urinary leakage (OR = 0.92; 95% CI: 0.71–1.20; *p* = 0.542).

When history of urinary leakage was examined as an exploratory outcome, weekly training hours showed a near-threshold association (OR = 0.67; 95% CI: 0.45–1.00; *p* = 0.051), and years of rugby practice showed a statistically significant association (OR = 0.74; 95% CI: 0.56–0.97; *p* = 0.028). BMI was not significantly associated with history of urinary leakage (OR = 0.84; 95% CI: 0.67–1.06; *p* = 0.145). However, these findings should be interpreted cautiously because training hours and years of practice are indirect indicators of sport exposure and do not capture intensity, playing position, contact load, impact frequency, match exposure, or rugby-specific lumbopelvic loading (Table 7).

## 4. Discussion

The present study examined ultrasound-derived transversus abdominis (TrA) thickness and thickness change using rehabilitative ultrasound imaging in nulliparous female rugby players and explored its relationship with urinary leakage. Overall, TrA thickness increased bilaterally during the abdominal drawing-in maneuver (ADIM), indicating that the ultrasound protocol was able to detect task-related thickness changes under standardized low-load conditions. However, static and semi-static ultrasound-derived TrA measures were not significantly associated with current urinary leakage. In addition, immediate ultrasound-based visual feedback did not produce a statistically significant additional increase in TrA thickness, and weekly training hours, years of rugby practice, and body mass index (BMI) were not significantly associated with current urinary leakage. These findings suggest that isolated TrA thickness assessment during controlled low-load tasks may not be sufficient as a standalone marker of urinary leakage status in female rugby players. From a functional morphology and kinesiology perspective, continence during rugby may depend more on dynamic coordination between the TrA, pelvic floor muscles (PFM), diaphragm, breathing strategy, and other lumbopelvic stabilizers than on muscle thickness alone.

### 4.1. Urinary Leakage in Female Rugby Players

One of the objectives of this study was to describe the prevalence and characteristics of urinary leakage in nulliparous female rugby players. In the present sample, 41.2% of participants reported having experienced urinary leakage at some point, while 20.6% reported current urinary leakage. These findings support the relevance of considering urinary leakage as a sports medicine and rehabilitation issue in women’s rugby, rather than only as a urogynecological condition. Previous studies have shown that urinary incontinence is common among physically active women and female athletes, especially in sports involving repeated impacts, jumps, landings, changes in direction, and increases in intra-abdominal pressure [2,8,11].

Among participants who reported the type of urinary leakage, stress urinary incontinence was the most frequent pattern. This interpretation should also consider that one participant who reported previous urinary leakage did not provide sufficient information to classify leakage type. This finding is consistent with the physiological mechanism proposed for high-impact and contact sports, in which repeated increases in intra-abdominal pressure may exceed the capacity of the PFM to maintain urethral closure during effort [5,25]. Rugby involves repeated acceleration, deceleration, sprinting, tackling, jumping, contact, trunk stabilization, and changes in direction. These demands may challenge lumbopelvic control and pressure regulation, particularly during high-intensity or fatigue-related situations. Therefore, even in nulliparous athletes, urinary leakage may appear as a functional manifestation of the interaction between sport-specific mechanical load and pelvic floor response.

The perceived impact of urinary leakage on daily life was low in this sample. This may indicate that symptoms were mild, but it may also reflect underreporting, normalization of symptoms, or adaptation strategies among athletes. Previous research has suggested that female athletes may downplay urinary leakage due to embarrassment, stigma, lack of information, or the belief that symptoms are an expected consequence of sport participation [21,30]. Therefore, a low perceived impact should not lead clinicians, coaches, or sports organizations to underestimate pelvic health concerns. Even mild leakage may affect confidence, hydration strategies, clothing choices, training behavior, or willingness to seek professional support.

### 4.2. Ultrasound-Derived TrA Thickness Response During the ADIM Task

A central objective of this study was to characterize ultrasound-derived TrA thickness and thickness change using rehabilitative ultrasound imaging under resting, ADIM, and visual-feedback conditions. The results showed a clear increase in TrA thickness during ADIM on both sides. This finding indicates that the protocol was able to identify task-related thickening of the deep abdominal wall in female rugby players.

The TrA is considered an important component of lumbopelvic stabilization, anticipatory trunk control, force transmission, and regulation of intra-abdominal pressure [13,19]. These functions are particularly relevant in rugby because the sport requires repeated trunk stiffness, pelvic control, acceleration and deceleration, physical contact, and rapid changes in direction. From this perspective, the observed increase in TrA thickness during ADIM suggests that rehabilitative ultrasound imaging may be useful for describing ultrasound-derived deep abdominal muscle thickness responses in athletes exposed to high lumbopelvic demands.

However, it is important to interpret this finding within the limits of the task used. ADIM is a standardized low-load motor task that can be used to examine TrA thickness change under controlled conditions. Although it is useful for assessing selective TrA thickening, it does not reproduce the dynamic, high-pressure, contact-related, and bracing-dependent conditions of rugby. Therefore, ultrasound-derived TrA thickening during ADIM should be interpreted as a structural thickness response during a controlled task, not as a direct measure of sport-specific trunk function, neuromuscular activation, or continence control during play.

This distinction is clinically relevant because abdominal bracing and ADIM represent different motor strategies. ADIM emphasizes selective drawing-in of the lower abdominal wall under low-load conditions, whereas abdominal bracing involves a broader co-contraction strategy aimed at increasing trunk stiffness. Rugby-specific actions such as tackling, contact preparation, sprinting, cutting, and resisting external forces are more likely to require bracing, anticipatory postural control, and rapid pressure regulation than isolated drawing-in. Therefore, the present findings should not be generalized to dynamic rugby-specific trunk stabilization tasks.

### 4.3. Association Between TrA Ultrasound-Derived Measures and Urinary Leakage

Another objective was to explore whether static and semi-static ultrasound-derived TrA measures were associated with urinary leakage. In the present study, current urinary leakage was not significantly associated with right-left TrA thickness difference, right TrA thickness, or left TrA thickness. These results suggest that TrA thickness alone may not be sufficient as a standalone ultrasound-derived marker of current urinary leakage status in this sample of female rugby players.

This finding is consistent with the concept that continence during sport depends not only on the structural characteristics of the TrA or PFM, but also on neuromuscular timing, coordination, pressure management, breathing strategies, and task-specific lumbopelvic control. Previous studies have emphasized the importance of functional synergy between the TrA and PFM in pelvic stability and urinary continence [5,9,18]. Therefore, a player may show adequate TrA thickening during a controlled ADIM task and still experience leakage during rugby-specific actions if the timing or coordination between the abdominal wall, diaphragm, and PFM is not sufficient to manage rapid increases in intra-abdominal pressure.

Importantly, PFM function was not directly assessed in this study. Therefore, any interpretation regarding TrA–PFM coordination should be considered mechanistic and hypothesis-generating rather than a measured finding of the present study. The absence of direct PFM assessment also means that pelvic floor strength, endurance, timing, relaxation capacity, and response to impact or fatigue could not be examined. Consequently, the present findings should be interpreted as describing ultrasound-derived TrA thickness responses, not the full continence mechanism.

The exploratory analysis using history of urinary leakage showed an association with right-left TrA thickness difference. However, this finding should be interpreted with caution due to the small sample size and the wide confidence interval. History of urinary leakage may reflect past episodes associated with specific training contexts, match situations, fatigue, or temporary physiological factors, whereas current urinary leakage reflects symptoms present at the time of assessment. For this reason, the main interpretation should be based on current urinary leakage, for which no significant associations with TrA thickness variables were observed. Future studies should distinguish between current leakage, lifetime history of leakage, and sport-specific leakage episodes.

### 4.4. Immediate Effect of Ultrasound-Based Visual Feedback

This study also examined whether immediate ultrasound-based visual feedback was associated with additional TrA thickness change during ADIM. The results showed that TrA thickness increased during voluntary activation, but the addition of real-time visual feedback did not produce a statistically significant additional increase in TrA thickness.

Our results should not be interpreted as evidence that ultrasound feedback is ineffective. Rather, it suggests that a single immediate exposure to visual feedback during a controlled low-load task may be insufficient to produce additional measurable changes in TrA thickness in trained rugby players.

Real-time ultrasound feedback has been proposed as a useful strategy to improve awareness and motor control of deep abdominal muscle behavior, especially in individuals with difficulty performing the ADIM task or with altered motor-control strategies [6]. However, rugby players may already have relatively developed trunk control due to the repeated stabilization demands of their sport. This could reduce the magnitude of the immediate observable effect of feedback during a simple task such as ADIM.

Therefore, the present findings suggest that the potential value of ultrasound-based feedback should be assessed in a different way. Rather than focusing only on immediate changes in TrA thickness during one controlled maneuver, future studies should evaluate whether repeated ultrasound-guided training improves TrA–PFM coordination, breathing strategies, lumbopelvic control, and urinary leakage symptoms during functional or sport-specific tasks.

### 4.5. Sport-Related Variables, BMI, and Urinary Leakage

A further objective was to explore whether training-related variables and BMI were associated with urinary leakage. Weekly training hours and years of rugby practice were not significantly associated with current urinary leakage. BMI was also not significantly associated with current urinary leakage. These findings should be interpreted cautiously because of the small sample size and the limited number of participants with current urinary leakage.

The absence of significant associations does not necessarily mean that training load or sport exposure are irrelevant. Weekly training hours and years of practice are indirect indicators and do not capture the real mechanical and physiological demands experienced by rugby players. In rugby, pelvic floor and lumbopelvic load may depend on playing position, number of tackles, contact exposure, sprint volume, jumping and landing frequency, strength training, fatigue, match intensity, and recovery status. This may explain why studies in female athletes show heterogeneous results regarding the relationship between sport exposure and urinary incontinence [10,26,29].

The lack of association between BMI and current urinary leakage is also plausible in this athletic population. In the general female population, BMI is commonly considered a risk factor for urinary incontinence. However, in rugby players, BMI is difficult to interpret because higher values may reflect greater lean mass rather than excess adiposity. In this context, BMI should not be interpreted as a direct proxy for adiposity or body composition, particularly in rugby players, whose anthropometric profiles may be influenced by lean mass and playing demands. In this athletic population, BMI should be interpreted with caution because it does not distinguish between fat mass and lean mass. This is particularly relevant in rugby players, whose anthropometric profiles may be strongly influenced by muscle mass and position-specific physical demands. Therefore, BMI alone may be insufficient to characterize the relationship between anthropometry, sport performance, and pelvic health in female rugby players. Future research should include body composition measures, such as fat mass, lean mass, and trunk muscle mass, together with sport-specific exposure variables, to better understand how physical profile and rugby-related loading interact with urinary leakage [20,22,29].

### 4.6. Practical Implications for Sports Rehabilitation and Athlete Monitoring

The findings of this study have practical implications for sports rehabilitation and athlete monitoring in women’s rugby. First, the presence of urinary leakage in a relevant proportion of players supports the inclusion of pelvic health screening in female rugby settings. Second, rehabilitative ultrasound imaging may be useful to assess TrA morphology and thickness changes during standardized tasks. Third, isolated TrA thickness should not be interpreted as a direct indicator of continence status. Instead, pelvic health assessment in rugby should consider dynamic coordination between the TrA, PFM, diaphragm, breathing pattern, and lumbopelvic control.

From an applied perspective, prevention and rehabilitation strategies should not focus exclusively on strengthening or increasing muscle thickness. They should also address motor control, motor timing, pressure management, breathing strategies, and sport-specific functional demands. Future assessment protocols should include dynamic tasks that resemble rugby actions, such as jumping, landing, sprinting, cutting, tackling preparation, resisted trunk actions, and fatigue conditions. Combining ultrasound imaging with pelvic floor assessment, electromyography, movement analysis, body composition assessment, and patient-reported outcomes may provide a more comprehensive understanding of continence mechanisms in female rugby players.

### 4.7. Strengths, Limitations and Future Research

This study has several strengths. First, it addresses an underexplored population of nulliparous female rugby players, a group exposed to high lumbopelvic and pelvic floor demands but rarely examined in pelvic health research. Second, the study combined urinary leakage assessment with bilateral rehabilitative ultrasound imaging of the TrA under standardized resting, ADIM, and visual-feedback conditions. Third, all ultrasound assessments were performed by the same physiotherapist, and three measurements were recorded for each condition, with the mean value used for analysis. Finally, the study distinguished between current urinary leakage and history of urinary leakage, which provides a more nuanced description of pelvic health symptoms in this athletic population.

Furthermore, this study has several limitations. No formal a priori sample size calculation was performed, and the final sample was determined by player availability. First, the cross-sectional design prevents causal or temporal interpretation of the observed associations. Second, the sample size was modest, and only seven participants reported current urinary leakage, limiting statistical power and the precision of the estimates. Third, participants were recruited through convenience sampling from a limited number of rugby clubs, which may restrict generalizability to other competitive levels or contexts. In addition, the number of players initially approached and the reasons for non-participation were not systematically recorded, preventing reconstruction of a detailed participant flow diagram. Fourth, urinary leakage was assessed using self-report, which may be influenced by recall bias, embarrassment, or underreporting.

Fifth, TrA assessment was performed during controlled static and semi-static tasks, not during dynamic rugby-specific actions. Although the ADIM task provides a standardized low-load assessment condition, it does not reproduce abdominal bracing or the high-pressure, contact-related, and fatigue-related demands of rugby. Respiratory instructions were standardized, but respiratory flow, breath-holding duration, and intra-abdominal pressure were not instrumentally monitored. Sixth, the study did not directly assess PFM function, TrA–PFM timing, intra-abdominal pressure, body composition, fatigue, playing position, or match exposure. Therefore, pelvic floor strength, endurance, relaxation capacity, and continence responses during sport-specific actions could not be directly examined. Finally, although ultrasound measurement was performed under standardized conditions and by the same physiotherapist, future studies should include intra-rater and inter-rater reliability data when possible.

Future research should use larger multicenter samples and longitudinal designs to examine whether ultrasound-derived TrA thickness responses, PFM function, respiratory control, intra-abdominal pressure regulation, and sport-specific load are associated with urinary leakage over time. Studies should also evaluate dynamic tasks that reproduce rugby demands and should examine whether ultrasound-guided interventions can improve TrA-PFM coordination, lumbopelvic control, and pelvic health outcomes in female rugby players. Future studies should also collect rugby-specific exposure variables, including playing position, match exposure, tackle/contact load, strength training, fatigue, menstrual status, hormonal contraceptive use, previous pelvic floor physiotherapy, and previous sports participation, as these factors may help explain interindividual differences in urinary leakage among female rugby players.

## 5. Conclusions

Urinary leakage was present in a relevant proportion of nulliparous female rugby players, with 41.2% reporting leakage at some point and 20.6% reporting current urinary leakage. Among participants who reported leakage type, stress urinary incontinence was the most frequent pattern, although the perceived impact on daily life was low.

Rehabilitative ultrasound imaging detected bilateral increases in TrA thickness during the ADIM task. However, static and semi-static ultrasound-derived TrA thickness measures, including side-to-side differences, were not significantly associated with current urinary leakage. Immediate ultrasound-based visual feedback did not produce a statistically significant additional increase in TrA thickness.

Weekly training hours, years of rugby practice, and BMI were not significantly associated with current urinary leakage. These findings suggest that isolated TrA thickness assessment during controlled low-load tasks may not be sufficient as a standalone marker of urinary leakage status in female rugby players.

Future research should include larger samples, direct pelvic floor muscle assessment, respiratory monitoring, body composition measures, and dynamic rugby-specific tasks to better understand continence mechanisms in women’s rugby.

## Figures and Tables

**Table 1:** Participant characteristics and sport-related profile.

Variable	Mean (SD)	Range
Age (years)	26.3 (5.0)	18.9–37.4
Weight (kg)	68.0 (10.4)	53–95
Height (m)	1.64 (0.05)	1.55–1.78
BMI (kg/m^2^)	25.2 (3.7)	21.0–35.8
Training hours/week	6.8 (3.2)	2–17
Years practicing rugby	7.4 (5.2)	1–27

Note: Where BMI, body mass index; kg, kilogram; m, meter; kg/m^2^, kilograms per square meter.

**Table 2:** Clinical and contextual characteristics of the participants.

Variable	Response	Freq.	Percent
Education level	High School/Technical	7	20.6
	University	27	79.4
	Student	13	38.2
Employment status	Employed	13	38.2
	Unemployed	2	5.9
	Student/Employed	6	17.6
Smoker	No	27	79.4
	Yes	3	8.8
	Former smoker	4	11.8
Constipation	No	25	73.5
	Yes	5	14.7
	Sometimes	4	11.8
Family history of urinary incontinence	No	17	50.0
	Yes	6	17.6
	Don’t know	11	32.3
Reproductive system anomalies	No	32	94.1
	Yes	2	5.9
Gynecological surgery	No	30	88.2
	Yes	4	11.8
Urinary tract infection in the previous month	No	33	97.1
	Yes	1	2.9

Note: *n*, number of participants; %, percentage; Freq., frequency.

**Table 3:** Prevalence and characteristics of urinary leakage.

Variable	Response	*n*	%
Ever experienced urinary leakage	No	20	58.8
	Yes	14	41.2
Current urinary leakage	No	27	79.4
	Yes	7	20.6
Frequency of current urinary leakage	1–3 times/week	6	85.7
	1 time/day	1	14.3
Amount of current urinary leakage	Very small amount	6	85.7
	Moderate amount	1	14.3
Type of urinary leakage reported	Stress urinary incontinence	9	69.2
	Mixed urinary incontinence	3	23.1
	Urgency urinary incontinence	1	7.7
Impact on daily life	1	27	79.4
	2	5	14.7
	3	1	2.9
	4	1	2.9

Note: Percentages for history of urinary leakage, current urinary leakage, and impact on daily life were calculated using the total sample as denominator (*n* = 34). Percentages for frequency and amount of current urinary leakage were calculated among participants with current urinary leakage (*n* = 7). Percentages for type of urinary leakage were calculated among participants who reported leakage type (*n* = 13). One participant who reported previous urinary leakage did not provide sufficient information to classify leakage type. In the administered questionnaire, the impact item was scored from 1 to 10, where 1 indicated no impact, and 10 indicated maximum impact.

**Table 4:** Ultrasound-derived TrA thickness at rest, during ADIM, and during ADIM with visual feedback.

Condition	Right TrA Thickness, cm	Left TrA Thickness, cm	Right-Left Difference, cm	*p*-Value for Right-Left Difference
Rest	0.42 ± 0.08	0.46 ± 0.10	−0.03 ± 0.07	0.008
ADIM without visual feedback	0.84 ± 0.19	0.80 ± 0.19	0.04 ± 0.18	0.241
ADIM with visual feedback	0.86 ± 0.19	0.85 ± 0.21	0.01 ± 0.16	0.603

Note: Data are presented as mean ± standard deviation. ADIM, abdominal drawing-in maneuver; TrA, transversus abdominis. Right-left differences were calculated as right minus left values.

**Table 5:** Immediate effect of ultrasound-based visual feedback on TrA thickness during ADIM.

Comparison	Mean Difference, cm	95% CI	*p*-Value
Right TrA: ADIM with feedback vs. ADIM without feedback	0.018	−0.059 to 0.096	0.639
Left TrA: ADIM with feedback vs. ADIM without feedback	0.041	−0.042 to 0.124	0.327

Note: ADIM, abdominal drawing-in maneuver; TrA, transversus abdominis. Where positive values indicate greater TrA thickness during ADIM with visual feedback compared with ADIM without visual feedback.

**Table 6:** Exploratory associations between TrA ultrasound-derived measures and urinary leakage.

Outcome	Predictor	OR	95% CI	*p*-Value
Current urinary leakage	Right-left TrA thickness difference	0.28	0.01–9.55	0.481
Current urinary leakage	Right TrA thickness	0.58	0.09–3.88	0.574
Current urinary leakage	Left TrA thickness	0.84	0.12–6.07	0.862
History of urinary leakage	Right-left TrA thickness difference	27.59	1.43–530.80	0.028
History of urinary leakage	Right TrA thickness	0.65	0.14–3.06	0.587
History of urinary leakage	Left TrA thickness	0.19	0.03–1.06	0.058

Note: CI, confidence interval; OR, odds ratio; TrA, transversus abdominis.

**Table 7:** Exploratory associations between sport-related variables, BMI, and urinary leakage.

Outcome	Predictor	OR	95% CI	*p*-Value
Current urinary leakage	Training hours/week	0.89	0.64–1.23	0.482
Current urinary leakage	Years practicing rugby	0.96	0.80–1.15	0.651
Current urinary leakage	BMI	0.92	0.71–1.20	0.542
History of urinary leakage	Training hours/week	0.67	0.45–1.00	0.051
History of urinary leakage	Years practicing rugby	0.74	0.56–0.97	0.028
History of urinary leakage	BMI	0.84	0.67–1.06	0.145

Note: BMI, body mass index; CI, confidence interval; OR, odds ratio. ORs are reported per one-unit increase in the corresponding predictor: one additional training hour per week, one additional year of rugby practice, or one additional kg/m^2^ for BMI. Analyses were exploratory and should be interpreted cautiously given the modest sample size.

## Data Availability

The datasets presented in this article are not readily available because the data are part of an ongoing study. In the meantime, additional materials and specific data supporting the findings of this study can be made available upon reasonable request to the corresponding author.
